# Identification and prioritization of novel therapeutic candidates against glutamate racemase from *Klebsiella pneumoniae*

**DOI:** 10.1371/journal.pone.0317622

**Published:** 2025-02-06

**Authors:** Ankit Kumar, Farah Anjum, Md Imtaiyaz Hassan, Anas Shamsi, Rashmi Prabha Singh

**Affiliations:** 1 Department of Biotechnology, Sharda School of Engineering and Technology, Sharda University, Greater Noida, Uttar Pradesh, India; 2 Department of Clinical Laboratory Sciences, College of Applied Medical Sciences, Taif University, Taif, Saudi Arabia; 3 Centre for Interdisciplinary Research in Basic Sciences, Jamia Millia Islamia, New Delhi, India; 4 Centre of Medical and Bio-Allied Health Sciences Research, Ajman University, Ajman, United Arab Emirates; 5 Department of Life Science, Sharda School of Basic Sciences and Research, Sharda University, Greater Noida, Uttar Pradesh, India; Chung-Ang University, REPUBLIC OF KOREA

## Abstract

**Background:**

*Klebsiella pneumoniae*, a gram-negative bacterium in the *Enterobacteriaceae* family, is non-motile, encapsulated, and a major cause of nosocomial infections, particularly in intensive care units. The bacterium possesses a thick polysaccharide capsule and fimbriae, which contribute to its virulence, resistance to phagocytosis, and attachment to host cells. The bacterium has developed serious resistance to most antibiotics currently in use.

**Objective:**

This study aims to investigate the structural properties of MurI (glutamate racemase) from *Klebsiella pneumoniae* and to identify potential candidate inhibitors against the protein, which will help in the development of new strategies to combat the infections related to MDR strains of *Klebsiella pneumoniae*.

**Methods:**

The 3D structure of the protein was modelled using SWISS-MODEL, which utilizes the homology modelling technique. After refinement, the structure was subjected to virtual high throughput screening on the TACC server using Enamine AC collection. The obtained molecules were then put through various screening parameters to obtain promising lead candidates, and the selected molecules were then subjected to MD simulations. The data obtained from MD simulations was then assessed with the help of different global dynamics analyses. The protein-ligand complexes were also subjected to MM/PBSA-based binding free energy calculation using the *g_mmpbsa* program.

**Results:**

The screening parameters employed on the molecules obtained via virtual screening from the TACC server revealed that Z1542321346 and Z2356864560 out of four molecules have better potential to act as potential inhibitors for MurI protein. The binding free energy values, which came out to be -27.26±3.06 kcal/mol and -29.53±4.29 kcal/mol for Z1542321346 and Z2356864560 molecules, respectively, favoured these molecules in terms of inhibition potential towards targeted protein.

**Conclusion:**

The investigation of MurI via computational approach and the subsequent analysis of potential inhibitors can pave the way for developing new therapeutic strategies to combat the infections and antibiotic resistance of *Klebsiella pneumoniae*. This study could significantly help the medical fraternity in the treatment of infections caused by this multidrug-resistant pathogen.

## 1. Introduction

*Klebsiella pneumoniae* is an opportunistic gram-negative bacterium belonging to the *Enterobacteriaceae* family. In 1882, Carl Friedlander first isolated *Klebsiella pneumoniae* from the lungs of a deceased human who suffered from pneumonia. The bacteria were described as non-motile and encapsulated, covered by a thick polysaccharide capsule, its essential virulence factor [1, 2]. This capsule prevents the bacteria from being phagocytosed. Fimbriae are another virulence factor that allows the bacteria to attach to the host cells [1].

*K*. *pneumoniae* is found predominantly on the mucosal surface, such as the nasal and gastrointestinal tract. The infection from these tracts can spread to the tissue and blood, leading to severe infection. Due to its high prevalence in ICU wards and other nosocomial infections and its increasing resistance to antibiotics, it has been included in the ESKAPE family [2]. Even with antibiotic discovery as early as Beta-lactam, *K*. *pneumoniae* successfully showed resistance due to the Beta-lactamase enzyme [1]. In 2013, out of 9,000 infections reported to the Centre for Disease Control and Prevention (CDC) due to carbapenem-resistant *Enterobacteriaceae*, 80% were due to *K*. *pneumoniae* [1]. In countries such as Singapore, the mortality rate due to *K*. *pneumoniae* is ranged from 20–26% [3]. The high prevalence and severity of infection caused by this pathogen have led to it being ranked in the critical priority list of the WHO pathogen priority list [4]. The Infectious Diseases Society of America has classified *K*. *pneumoniae* in the top six virulent multidrug-resistant (MDR) microorganisms in hospitals worldwide [5, 6]. The ability of a pathogen to acquire foreign DNA helps it to become multi-drug resistant. Succession of the pathogen has led to the development of two distinct types of strains, classified as classical *K*. *pneumoniae* (cKp) and hypervirulent *K*. *pneumoniae* (hvkp) [7]. The cKp is found as a nosocomial infection in North America and Europe, while the hvKp infects the community around the Asian Pacific rim and has also started to spread globally [8, 9]. The cKp strains are responsible for 14%-20% of respiratory, urinary, and surgical wound infections in the health care setting. The MDR and XDR variants are resistant to a broad spectrum of antibiotics, including β–lactam, carbapenems, fluoroquinolones, sulfonamides, and aminoglycosides. The new hvKp strains can cause severe infection in lower doses and infect all age groups [10]. The infectious syndromes can be highly diverse and spread over different body parts. Some examples include non-hepatic abscesses, pneumonia, necrotizing fasciitis, endophthalmitis, and meningitis [11]. The latest WHO report published in July 2024 revealed that India has reported the presence of hypervirulent hvKp variants [12]. A study conducted to see the trends in the antibiotic resistance pattern of *Klebsiella pneumoniae* in North India revealed the resistance rate for *K*. *pneumoniae* was 7.5% in 2018, which increased to 21.4%. The cases of XDR *Klebsiella pneumoniae* in patients on ventilators increased from 62.5% in 2018 to 71% in 2022 [13].

Peptidoglycan serves as a crucial component of the bacterial cell wall, providing structural support that allows bacteria to counter adverse environmental conditions. Moreover, it plays a vital role in maintaining cell shape as well as facilitating proper growth and replication [14]. Peptidoglycan is a heteropolymer consisting of straight glycan chains with alternate repeating units of N-acetylglucosamine (GlNAc) and N-acetylmuramic acid (MurNAc) linked in beta 1–4 linkage [15]. The glycan chains are linked with a set of stem peptides comprising L-alanine (L-Ala), D-glutamate (D-Glu), D-alanine (D-Ala), and either L-lysine or 2,6-diaminopimelic acid. These stem peptides are covalently attached to MurNAc, forming a complex cellular framework. The inclusion of D-Glu as the second residue in the stem peptide is a conserved feature, making D-Glu essential for peptidoglycan biosynthesis. Intracellular D-Glu is produced through the racemization of L-glutamate (L-Glu). The enzymatic mechanism responsible for the production of D-glutamic acid in bacteria has been studied for a limited number of species [16]. Two distinct pathways have been identified for the biosynthesis of D-glutamic acid: (A): -Transamination Process: In certain bacteria, such as *Bacillus* species, D-glutamic acid is produced through a transamination process. This involves the catalysis of D-alanine and α-ketoglutarate by a D-amino acid transaminase, and (B):- Direct Conversion: The second route of D-glutamic acid biosynthesis involves a direct conversion of L-glutamic acid to D-glutamic acid facilitated by an enzyme Glutamate racemase (MurI). It has two domains over a pseudo-symmetric axis with a conserved topology [17]. The catalytic ‘racemization’ reaction, which employs a general acid-base mechanism, takes place at the deep pocket, flanked on both sides by a carboxy and amino-terminal domain. Structural analysis of MurI from *H*. *pylori* indicates coordinated hinge movement occurring at the crossover between these domains, which is essential for the formation of an active site aiding catalysis. The catalytic active site could be identified at the interface of the two domains, wherein the MurI conserved residues from the amino terminus domain (Asp7, Ser8, Cys70, Thr72) are usually associated with deprotonation of D-Glutamate and the carboxy terminus residues (Glu150, Cys181, Thr182, His183) are involved in L-Glutamate deprotonation [18].

Glutamate racemases share a common ‘two base racemization mechanism’ [19] that employs two cysteine residues at its active site, where one of these two deprotonates the substrate at the alpha position, and the other protonates the resulting carbanionic intermediate on the opposite face [20, 21].

The studies support MurI as a potential druggable target for a structure-based drug discovery approach as it is involved in the phase 1 peptidoglycan synthesis pathway [22]. Since this protein is actively involved in peptidoglycan synthesis by providing D-glutamate via racemization of L-glutamate, inhibiting this protein may hamper the peptidoglycan synthesis pathway in *Klebsiella pneumoniae*. In search of potential candidate inhibitors against glutamate racemase, a cryptic allosteric pocket near the hinge region between two domains of the protein was targeted. The involvement of this site in the non-competitive inhibition of MurI protein is explained by Chheda et al. in their study aimed to discover the role of this cryptic pocket for drug development and discovery against *H*. *pylori* targeting glutamate racemase. They discovered that the following residues of this particular cryptic allosteric pocket were found to interact with the ligand used for the study: Trp244, Gln248, Trp252, Val104, Glu150, His183, Ile49, Leu154, Leu186 [23]. Our study involves the investigation of the structural properties of MurI of *Klebsiella pneumoniae* (strain 342) via 3D-structure prediction using a homology modelling approach. The modelled structure will be used for high throughput virtual screening to find candidate inhibitor molecules, followed by molecular dynamics simulations. Global and essential dynamics analyses will be performed to assess the effects of ligand binding on the structural and functional properties of the MurI protein. This study will facilitate the development of candidate inhibitors to suppress the virulence mechanism of *Klebsiella pneumoniae*.

## 2. Methodology

### 2.1. Structure prediction and energy minimization

The amino acid sequence of MurI protein in FASTA format from *Klebsiella pneumoniae* (strain 342) was obtained from the UniProt database (Uniprot ID: B5XZ09) [24], then, BLASTp [25] was performed against Protein Data Bank (PDB) database [26] to identify the template for homology modelling. The template selection process relied on (a) the highest sequence identity, (b) ensuring sufficient query coverage, (c) considering the family of the protein, and (d) assessing the crystal structure resolution. After retrieving the template structure from RCSB-PDB [26], it was prepared before using it for homology modelling. In this process, any molecules bound to the protein were removed, and water molecules were also separated from the protein structure. The corrected template structure was then saved in PDB format. The MurI FASTA sequence from *Klebsiella pneumoniae* and the PDB file of the template protein structure were then submitted as inputs for 3-D structure prediction on the SWISS-MODEL server [27].

SWISS-MODEL employs a homology modelling approach to predict the structure of a protein. It utilizes two search methods for template selection: (1) BLAST for closely related templates and (2) HHblits for detecting remote homologs. In the process of building a model, coordinates for conserved atoms are first transferred based on the template-target alignment. The coordinates for insertions or deletions are generated with the help of loop modelling, and the non-conserved side chains are generated to complete the model. SWISS-MODEL utilizes the ProMod3 modeling [28] engine and OpenStructure framework [29] to build the models, and uses the QMEAN scoring function for the quantification of modeling errors and assessment of model accuracy [27]. The model obtained from this server was then subjected to model quality assessment followed by model refinement. For energy minimization and geometrical optimization of the modelled structure, GalaxyRefine2 [30] web server was utilized. GalaxyRefine2 estimates the residue-wise error of the input structure based on three scores: RMSF, FRAG, and MSA. RMSF calculates fluctuations in the structure during MD relaxation; FRAG evaluates the alignment of backbone torsion angles with fragment library angles; and MSA derives a score from multiple sequence alignment of homologous sequences. A linear model combines these scores to predict residue-wise error, marking regions with high error estimates as unreliable local regions (ULRs) [30]. After employing the energy minimization protocols on the initial model, one model out of the ten models provided by Galaxyrefine2 was chosen by considering the lowest RMSD and clash score values, as well as appreciable Ramachandran plot statistics.

### 2.2. Model evaluation and targeted site analysis

The model selected after energy minimization was then re-evaluated with the help of specific programs on the Structure Analysis and Verification Server (SAVES) platform (accessible at https://saves.mbi.ucla.edu/). Among the suite of tools offered by this server, emphasis was placed on PROCHECK [31], ERRAT [32], and VERIFY 3D [33, 34] for assessing model quality. PROCHECK evaluates protein structural stereochemistry by considering parameters like Phi, Psi, and Chi torsional angles, energies associated with H bonds, and distance between two bonded atoms in line with the Ramachandran plot. ERRAT discerns accurately resolved fragments from erroneously modelled ones by analyzing atomic interactions using statistical methods. VERIFY 3D establishes links between the 1D amino acid sequence and the 3D structure of a protein. The model assessment results for the initial model and refined model were then compared to assess the improvements that happened in the structure. In the next step, the allosteric cryptic site of the MurI protein, which was chosen for this study, was carefully analyzed. The crucial residues involved in the ligand binding to the targeted site were selected after carefully inspecting the respective site in the template and other closely related protein structures. The critical and conserved residues of the targeted cryptic pocket were identified with the help of pairwise sequence alignment between MurI from *K*. *pneumoniae* and MurI from *H*. *pylori* (PDB ID: 2JFX).

### 2.3. Virtual screening for the search for putative inhibitors

For the identification of candidate inhibitor molecules against the targeted cryptic site of the MurI protein, we performed virtual high throughput screening on the Discovery@TACC web portal (http://drugdiscovery.tacc.utexas.edu/). The screening was conducted against the Enamine AC library in a site-specific manner, targeting an allosteric cryptic pocket of the enzyme. Using PyRx [35] software, we constructed a grid box tailored to this site, with dimensions (Å) set as X: 66.19, Y: 64.97, Z: 57.63, and coordinates (centre) X: 37.42, Y: 24.75, Z: 33.29. The refined predicted structure for MurI in PDBQT format and the coordinates for the targeted site were then submitted to the TACC portal. The TACC portal utilizes Auto Dock Vina [36] to find the molecules having high binding affinities for the targeted site of the protein. The default exhaustiveness value is 8, and the energy range is 4 for the virtual screening on this server. Finally, the TACC portal provides 1000 molecules possessing the best binding affinities for the targeted site.

### 2.4. Pharmacokinetics and drug likeness

In the pursuit of candidate inhibitor molecules, the initial pool of 1000 compounds, obtained through virtual screening via the TACC server, underwent further assessment using the DataWarrior [37] tool. The DataWarrior program screens molecules based on the following parameters: Molecular weight, cLogP, H-acceptors, H-donor, TPSA, Druglikeness, and rotatable bond counts. In addition to Lipinski’s rule of five, other filters were applied to gauge toxicity, cancer-inducing potential, effects on reproductive health, mutagenicity, and irritability. After applying these filters, roughly half of the molecules were eliminated. The remaining compounds then underwent screening on the SwissADME server [38], focusing on ADME (adsorption, distribution, metabolism, and excretion) parameters; only molecules meeting these criteria progressed further. Subsequently, four compounds were selected based on the best estimated binding affinity values.

### 2.5. Molecular dynamics simulations

Next, molecular dynamics (MD) simulations were conducted with the help of the GROMACS 2021.3 [39] package was utilized. The ligand topologies were initially prepared using the SwissParam web server [40, 41]. Subsequently, 100-nanosecond MD simulations were carried out in triplicates for both the native protein and its ligand-bound complexes, with the CHARMM27 [42] force field employed to parameterize the MurI model. A cubic unit cell with a d-value of 1.0 was defined for the simulation. The protein system was then solvated using the TIP4P water model. The charge on the protein molecule was then neutralized with the help of the *gmx genion* module of GROMACS, which replaces water molecules with appropriate counter ions. The native MurI and its complexes with the ligands then underwent energy minimization, for which the steepest descent algorithm was used, with a maximum force threshold of 100 kJ/mol/nm. The system underwent a two-step equilibration process: first, a 3-nanosecond NVT equilibration at constant pressure and volume, followed by a 3-nanosecond NPT equilibration at constant pressure and temperature. Both equilibration phases comprised 15,00,000 steps each. Finally, 100-nanosecond production MD simulations were performed with a time step of 2 femtoseconds. Various commands within the GROMACS 2021.3 package were used to analyze the MD trajectories. Key Parameters analyzed for unbound protein and protein-ligand complexes include RMSD, RMSF, SASA, buried SASA, radius of gyration, and principal component analysis.

### 2.6. MM/PBSA binding free energy calculations

To elucidate the behaviour and interactions of ligands with the MurI protein and to calculate the associated interaction energies, the MM/PBSA (Molecular Mechanics/Poisson-Boltzmann Surface Area) method was employed [43]. The binding free energy (ΔG_binding_) of the MurI-ligand complexes was determined using the following equation:

$$\Delta G_{binding}=\Delta E_{vdw}+\Delta E_{ele}+\Delta G_{pol}+\Delta G_{np}$$ (1)

In this equation, ΔG_binding_ denotes the binding free energy, which is the sum of the changes in Van der Waals energy *(ΔE*_*vdw*_*)*, electrostatic energy (*ΔEele*), polar solvation energy (*ΔGpol*), and non-polar solvation energy (*ΔGnp*). To enhance the reliability and robustness of the analysis and to further bolster the statistical confidence, MM/PBSA was done in triplicates for each complex. Multiple runs also help minimize the effect of random errors and better understand the variations occurring in the results. The consistency in results from multiple runs also validates the methods used for calculations. The MM\PBSA-based binding free energy calculations were performed using entire trajectories for 0 to 100 ns, and after every 100 ps, a snapshot of protein-ligand complexes was taken, and the analysis was performed in triplicates.

## 3. Results

### 3.1. Homology modelling and energy minimization

The absence of human homologs for the MurI protein of *K*. *pneumoniae* was confirmed through a similarity search using BLASTp against the human protein sequence database. For homology modelling, the SWISS-MODEL server was utilized for predicting the 3D structure of the MurI protein from *K*. *pneumoniae*, and the crystal structure of MurI (*E*. *coli)* with PDB ID: 2JFN was taken as a template. The obtained model from the SWISS-MODEL server was then subjected to model quality assessment using the SAVES server, followed by refinement using the GalaxyRefine2 server. Out of 10 models provided by this server, on the basis of clash score and Ramachandran values, one model was selected which was again subjected to model quality assessment. The model quality scores before and after energy minimization are provided in Table 1.

**Table 1:** Model quality assessment scores before and after energy minimization based on different programs on SAVES server.

S.No.	Model	Potential Energy (kJ/mol)	ERRAT Quality Factor.	PROCHECK				Verify-3D
				Residues in most favoured regions	Residues in additional allowed regions	Residues in generously allowed regions	Residues in disallowed regions	
**1.**	Initial model	-119940.8	99.60	94.3%	5.7%	0%	0%	98.85% (Pass)
**2.**	Refined model	-155916.7	100	96.0%	4%	0%	0%	98.47% (Pass)

### 3.2. Model evaluation and binding site analysis

The minimized model was assessed using the SAVES server (https://saves.mbi.ucla.edu/) and compared with the initial model. Evidently, the minimized model exhibited improved scores across all assessment parameters, including ERRAT, VERIFY 3D, and PROCHECK. The potential energy of the initial model was -119940.8 kJ/mol, but after refinement, it was reduced to -155916.7 kJ/mol. The ERRAT score of the model also improved from 99.60 to 100, and both models passed the assessment via the Verify-3D program. The Ramachandran plot generated through the PROCHECK program revealed that 94.3% of residues of the initial model were in the most favoured regions. After energy minimization, they were recorded to be 96.0%. The residues in additionally allowed regions for the initial and energy minimized model were 5.7% and 4% respectively. No residues were found in generously allowed or disallowed regions for both models; the values obtained from different model assessment parameters before and after energy minimization are presented in Table 1. After careful examination and pairwise sequence alignment, the following residues were found important and conserved: Leu172, His206, and Leu269. Additionally, Val111, Gly112, and Val113 residues, which were responsible for hinge movement, were also found to be conserved.

### 3.3. Virtual high-throughput screening and pharmacokinetics and drug-likeness

The screening performed on the DrugDiscovery@TACC portal yielded 1000 molecules with the highest binding affinities for the MurI protein. These molecules were further screened based on Lipinski’s rule of five, utilizing the DataWarrior tool. Out of 1000 initial candidates, 483 molecules met Lipinski’s criteria, exhibiting positive drug-likeness values and passing additional filters for toxicity, carcinogenicity, mutagenicity, irritability, and reproductive effects. These molecules were then subjected to ADME (absorption, distribution, metabolism, and excretion) screening using the SwissADME server. To facilitate this, molecules were converted from PDB format to SMILES using OpenBabel version 2.3.1. Ultimately, 198 out of the 483 molecules passed the ADME filters, which included checks for PAINS (Pan-assay interference compounds) alerts, gastrointestinal absorption, blood-brain barrier permeability, aqueous solubility, topological polar surface area (TPSA), and Cytochrome P inhibition.

The selected molecules demonstrated high gastrointestinal (GI) absorption levels and had a topological polar surface area (TPSA) of less than 100 Å^2^. Additionally, none of these molecules were identified as inhibitors of the cytochrome enzymes CYP1A2, CYP2C19, CYP2C9, CYP2D6, or CYP3A4, and they did not trigger any PAINS (Pan-assay interference compounds) alerts. All 198 molecules were found to be capable of crossing the blood-brain barrier and exhibited high solubility in an aqueous environment, with a cLogP value of less than 5. Ultimately, only 4 molecules were selected for further studies based on their estimated binding affinities and orientation towards the targeted site of the MurI protein. The binding affinity values offered by each ligand are -8.5 kcal/mol, -8.5 kcal/mol, -8.7 kcal/mol, and -8.8 kcal/mol for Z1542321346, Z2356864560, Z2228760853, and Z3214698999 molecules. The physiochemical properties, toxicological characteristics, and ADME characteristics of the selected ligands are provided in Tables 2–4. The allergenicity score for the final four ligands was also calculated using the ChAIpred server, and none of the molecules were found to be allergenic. The results for this analysis are provided in S1 Table, and further details for this analysis are also provided in the same file.

**Table 2:** Physiochemical properties of the screened ligands based on Lipinski’s rule of five.

S.No.	Molecule	MW (g/mol)	r-Bonds	HBD	HBA	Druglikeness
1.	Z1542321346	434.69	4	4	5	2.13
2.	Z2356864560	406.63	4	3	5	-0.50
3.	Z2228760853	458.61	6	3	5	-8.30
4.	Z3214698999	361.45	5	2	5	-5.43

Molecular weight **(MW),** Rotatable bonds **(r-Bonds),** Hydrogen Bond Donor **(HBD),** and Hydrogen bond acceptor **(HBA).**

**Table 3:** Toxicological characteristics of screened molecules.

S.No.	Molecule	Mutagenic	Tumorigenic	Reproductive effective	Irritant
1.	Z1542321346	None	None	None	None
2.	Z2356864560	None	None	None	None
3.	Z2228760853	None	None	None	None
4.	Z3214698999	None	None	None	None

**Table 4:** ADME profile of the selected ligands.

S.No.	Molecule	GI Absorption	BBB Permeability	cLogP	TPSA(Å^2^)	ESOL Class	CYP Inhibitor	PAINS Alert	Pgp substrate
1.	Z1542321346	High	Yes	3.39	67.76	Moderately Soluble	No	0	No
2.	Z2356864560	High	Yes	2.46	70.75	Moderately Soluble	No	0	Yes
3.	Z2228760853	High	Yes	3.74	50.77	Moderately Soluble	No	0	Yes
4.	Z3214698999	High	Yes	3.62	56.17	Moderately Soluble	No	0	Yes

Gastrointestinal Absorption (**GI**), Blood-Brain Barrier (**BBB**), Total Polar Surface Area (**TPSA**), Estimated Solubility (**ESOL**), Cytochrome P450 enzymes (**CYP**) and Pan-assay interference compounds (**PAINS),** ConsesuslogP (**ClogP**) P-glycoprotein (**Pgp**).

### 3.4. Protein-ligand interaction studies

**Fig 1:**
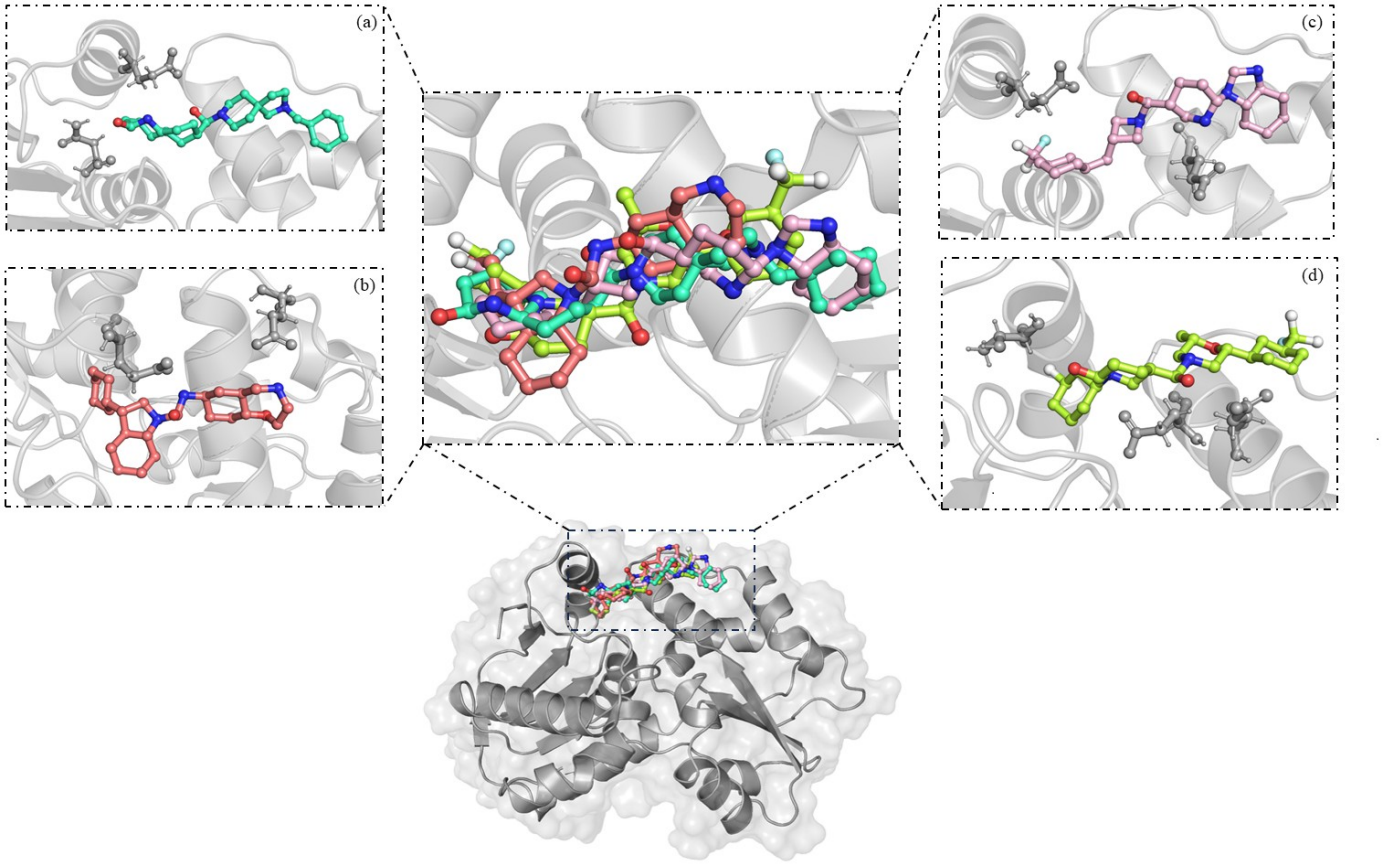
Binding of selected molecules to the targeted site of MurI. Three-dimensional Zoomed-in view: **(a)** Z1542321346 (Green cyan), **(b)**. Z2356864560 (Salmon), **(c)** Z2228760853 (Light pink), and **(d)** Z3214698999 (Limon). The protein residues involved in hydrogen bond formation with respective ligands are shown using ball and stick representation.

The interactions between protein and ligands were studied with the help of BIOVIA Discovery Studio. Fig 1 depicts the binding of the ligands to the targeted site of the protein, and it also shows the orientation of the ligand in the targeted site.

**Fig 2:**
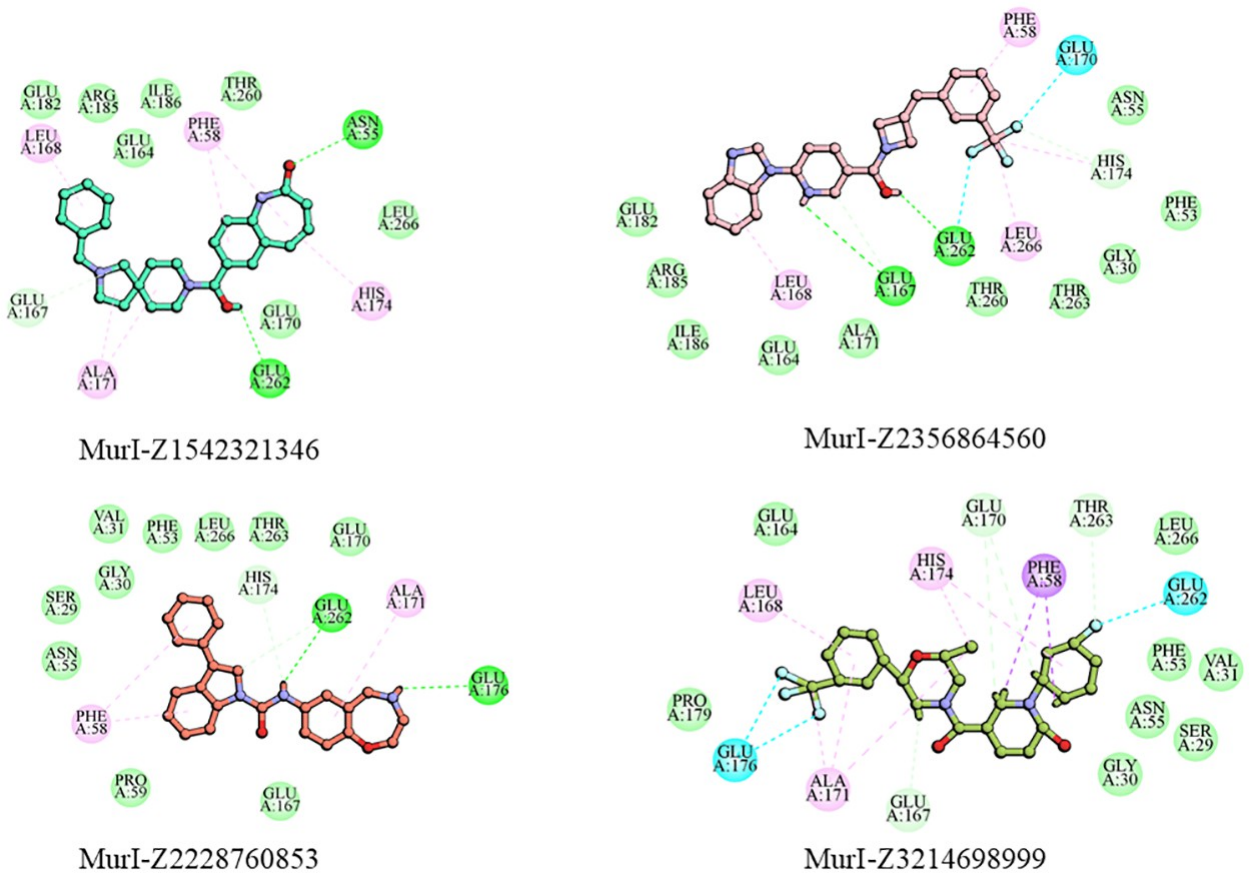
Two-dimensional representation of ligand’s interactions with the protein. Colour scheme: Hydrogen bonds (Green), Alkyl interactions (Pink), Pi-Alkyl interactions (Blue), Pi-Pi stacked interactions (Magenta), Pi-sigma interactions (Purple), Halogen interaction (Cyan).

The Z1542321346 ligand was found to form two hydrogen bonds with Asn55 and Glu252, and it was also involved in Pi-alkyl interactions with residues Leu168, Phe58, His174, and Ala171. Ligand also interacted via Van der Waals interaction with the protein involving residues Glu182, Arg185, Ile186, and Thr260, and a C-H bond was formed with Glu167 (Fig 2). The Z2356864560 ligand formed two hydrogen bonds with Glu176 and Glu262 residues. The ligand was also seen forming alkyl interactions with Ala 171 and two pi-alkyl interactions with Phe58. The amino acid residues taking part in Van der Waals interaction with this ligand are Ser29, Gly30, Phe53, Asn55, Pro59, Glu167, His174, Thr263, and Leu266 (Fig 2).

The MurI-Z2228760853 complex was stabilized with the help of two hydrogen bonds involving Glu167 and Glu262 residues. The Z2228760853 ligand also participated in alkyl hydrophobic interaction with Leu168 and Leu266 and also formed Pi/alkyl hydrophobic interaction with Phe58 and His174. The Van der Waals interactions were formed with residues Gly30, Phe53, Asn55, glu164, Ala171, Glu182, Arg185, Ile186, thr260, Thr263, and His174. Halogen interactions were also observed between the fluorine atoms of the ligand and Glu170 and Glu262 residues of the MurI protein (Fig 2).

The Z3214698999 molecule formed halogen interactions with the Glu176 and Glu262 residues through its three fluorine atoms. It was also involved in Pi-sigma interactions with residue Phe58 and formed Pi-alkyl interactions with His174. Its involvement in the formation of alkyl hydrophobic interactions was seen with Leu168 and Ala171 amino acid residues. Ligand took part in Van der Waals interactions with residues Ser29, Gly30, val31 Phe53 Asn55, Glu164, Glu167, Glu170, Thr263, and Leu266 (Fig 2). Table 5 depicts the type of interactions occurring between ligands and protein molecules along with the respective bond lengths. The common residues of the targeted site that were found to be interacting with all the ligands were Glu262 and Glu176, which were forming hydrogen bonds. Leu168, His174, Phe58, and Ala171 participated in pi interactions, and Glu167, Leu266, Phe53, Asn55, Thr263, and Thr260 were taking part in Van der Waals interactions.

**Table 5:** Interaction shown by selected ligands with MurI protein.

S.no	Ligands	HB	D (Å)	Pi-SR alkyl interactions	D (Å)	Van der Waals interactions	Halogen interaction	D (Å)
1	Z1542321346	Asn55 Glu262	2.37 2.50	Leu168 Phe58 His174 Ala171	4.23 4.57, 5.39 5.31 5.04, 5.11	Glu164 Glu167 Glu182 Arg185 Ile186 Thr260 Leu266		
2	Z2356864560	Glu262 Glu176	3.54, 2.48 2.19	Ala171 Phe58	4.52 4.61, 5.46	Ser29 Gly30 Phe53 Asn55 Pro59 Glu167 Glu170 His174 Thr263 Leu266		
3	Z2228760853	Glu167 Glu262	2.17, 3.01,	Leu168 Leu266 Phe58 His174	4.36 5.11 4.75 5.23	Gly30 Phe53 Asn55 Glu164 Ala171 Glu182 Arg185 Ile186 Thr260 Thr263 His174	Glu170 Glu262	3.17 3.64
4	Z3214698999			Ala171 Phe58 His174 Leu168	4.11, 4.40, 4.35 2.88, 2.90 3.77, 5.50 5.35	Ser29 Gly30 Val31 Phe53 Asn55 Glu164 Glu167 Glu170 Thr263 Leu266	Glu176 Glu262	3.20, 3.19 3.30

Distance **(D)**

**Fig 3:**
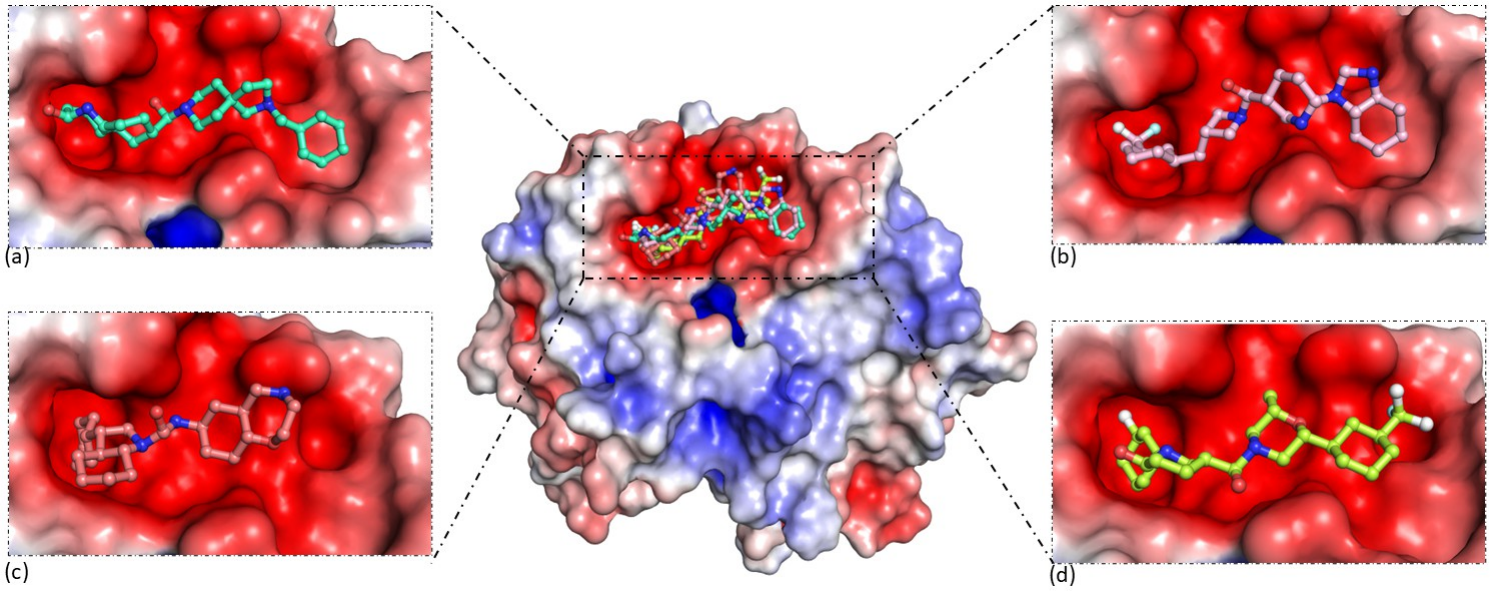
Three-dimensional Electrostatic surface potential maps of targeted cryptic allosteric pocket with bound ligands (a) Z1542321346 (Green cyan), **(b)**. Z2356864560 (Salmon), **(c)** Z2228760853 (Light pink), and **(d)** Z3214698999 (Limon).

The APBS plugin in PyMol was utilized to generate the electrostatic surface potential map for protein-ligand complexes. The red colour for the targeted site indicates that the site is acidic, which could be due to the presence of glutamate, glutamine, and asparagine amino acids. The lead molecules were seen to fill the targeted site’s groove effectively and were appropriately embedded in the groove (Fig 3).

### 3.5. Molecular dynamics simulations

Next, molecular dynamics simulations were performed for the native MurI protein and its ligand-bound complexes. The virtual screening techniques are effective for examining libraries containing a large number of molecules and finding out the best conformations of ligands with the target protein. However, molecular dynamics simulations are the only tool to accurately mimic the physiological conditions in which the ligand and protein molecules interact with each other.

In molecular dynamics simulations, both the ligand and the protein molecules are considered flexible entities, allowing them to undergo necessary structural changes to enhance interactions between them. However, positional restraints are applied to the protein and ligand molecules during the equilibration steps to avoid extravagant rearrangements in their structures because of the large forces in play. Moreover, MD simulations take into account physiological conditions such as temperature, pressure, aqueous environment, and ions to neutralize the system, providing a more realistic understanding of protein-ligand interactions. The simulations for both the protein and its complexes with selected molecules were conducted for 100 ns in triplicates.

#### 3.5.1. Root mean square deviation

**Fig 4:**
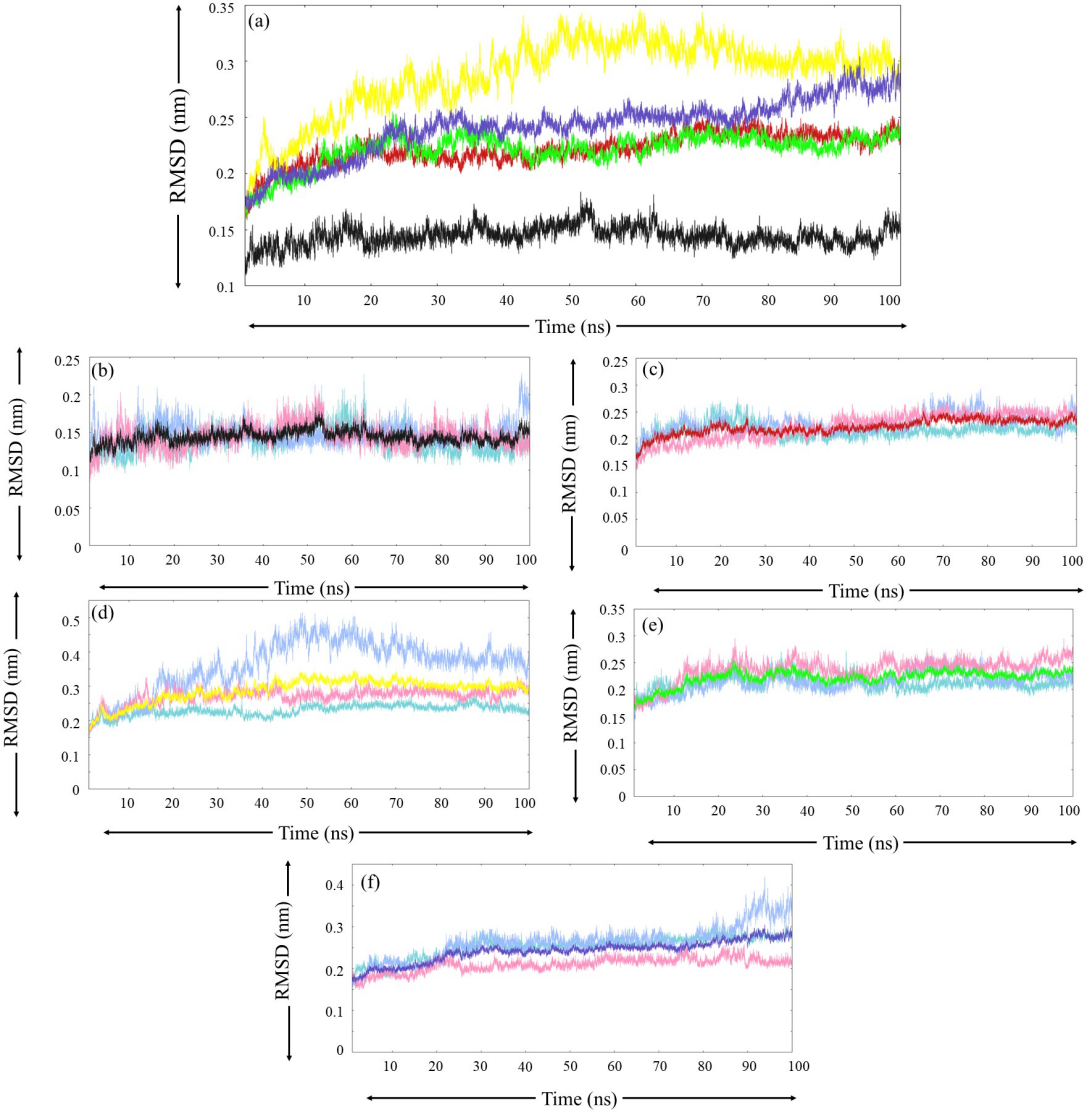
RMSD analysis (a) average RMSD plot for each protein system obtained from three MD simulation runs, colour-coded as black, red, yellow, green, and blue for the following protein system respectively (b) Free-MurI, (c) MurI-Z1542321346, (d) MurI-Z2356864560, (e) MurI- Z2228760853, and (f) MurI- Z3214698999. The RMSD trend for run-1, run-2, and run-3 are represented with turquoise, light blue, and pink colours, respectively.

RMSD analysis, when performed, revealed that the path taken by trajectories in run-1 and run-2 for native MurI protein was quite similar (Fig 4b). In both runs, the trajectories seem to converge after 70 ns. For run-3, the RMSD plot appeared to be linear without many fluctuations, but high RMSD values were recorded at the end of the simulation. The average RMSD values for native MurI protein during MD triplicates were recorded as 0.140 nm, 0.148 nm, and 0.144 nm, indicating low conformational variability for free protein during the simulations. The visual interpretation of the RMSD plot for the MurI-Z1542321346 complex (Fig 4c) revealed that there was a slight variation in the path followed by triplicate trajectories, but the average values were significantly closer, recorded as 0.214 nm, 0.227 nm, and 0.222 nm for run-1, run-2, and run-3 (Table 6). RMSD trajectories for run-1 and run-2 followed a similar path till 64.9 ns, after which the trajectory for run-2 appeared more perturbed. In run-3, dynamic equilibrium was attained at 45 ns, after which the RMSD value hovered around 0.24 nm. This complex displayed decent conformational stability during all three MD runs.

**Table 6:** Average value of different global analyses performed for MurI and its complexes.

RMSD (nm)					
	Native	Z1542321346	Z2356864560	Z2228760853	Z3214698999
run-1	0.140	0.214	0.229	0.211	0.252
run-2	0.148	0.227	0.360	0.214	0.262
run-3	0.144	0.222	0.270	0.237	0.209
Average	0.144	0.221	0.286	0.221	0.241
**RMSF (nm)**					
run-1	0.116	0.117	0.125	0.125	0.126
run-2	0.117	0.127	0.116	0.116	0.140
run-3	0.113	0.134	0.132	0.131	0.112
Average	0.115	0.126	0.124	0.124	0.126
**Rg (nm)**					
run-1	1.887	1.888	1.897	1.887	1.896
run-2	1.885	1.893	1.918	1.882	1.901
run-3	1.878	1.886	1.910	1.899	1.880
Average	1.883	1.889	1.908	1.889	1.892
**SASA (nm** ^**2**^ **)**					
run-1	131.56	130.33	131.92	132.78	132.24
run-2	129.80	132.82	134.63	131.01	135.58
run-3	129.17	131.28	133.78	131.92	129.81
Average	130.18	131.48	133.44	131.90	132.54

MurI-Z2356864560 complex exhibited noticeably stable graphs for run-1 and run-3; very few fluctuations were seen in the RMSD plot for these two runs. The plot for run-2 appeared highly perturbed; also, the RMSD values offered by this complex were quite high. (Fig 4d) The RMSD graphs obtained after MD triplicates for MurI-Z2228760853 displayed a substantial amount of consistency with slight deviations (Fig 4e). Till 50 ns of the simulation period, RMSD trajectories for all three MD runs followed a similar path, after which the graph for run-1 appeared to converge with the lowest RMSD among the triplicates, the graph for run-2 after exhibiting significant undulations converged to a similar point as run-1, whereas, for run-3 the graph attained stability after 60 ns but maintained a slightly higher RMSD value in comparison to other two runs. The average RMSD values for this complex were recorded to be 0.211 nm, 0.215 nm, and 0.237 nm for run-1, run-2, and run-3, respectively (Table 6).

The MurI- Z3214698999 complex displayed noteworthy consistency in RMSD results; for run-1 and run-2, the RMSD plots exhibited substantial resemblance till 86 ns, after which the plot for run-2 started to ascend and finally converged to a value of 0.34 nm (Fig 4f). The observed pattern of the graph for run-3 was also a lot like the other two runs, but the RMSD value steadily remained below 0.22 nm throughout the simulation period. The average values were recorded as 0.252 nm, 0.262 nm, and 0.209 nm for run-1, run-2, and run-3, respectively. The orderliness observed in the RMSD plot for the MurI-Z3214698999 complex during MD triplicates indicates good stability and lower conformational variability of the protein-ligand complex; these observations further backed up the robustness and reliability of the MD simulations.

#### 3.5.2. Root-mean square fluctuations

**Fig 5:**
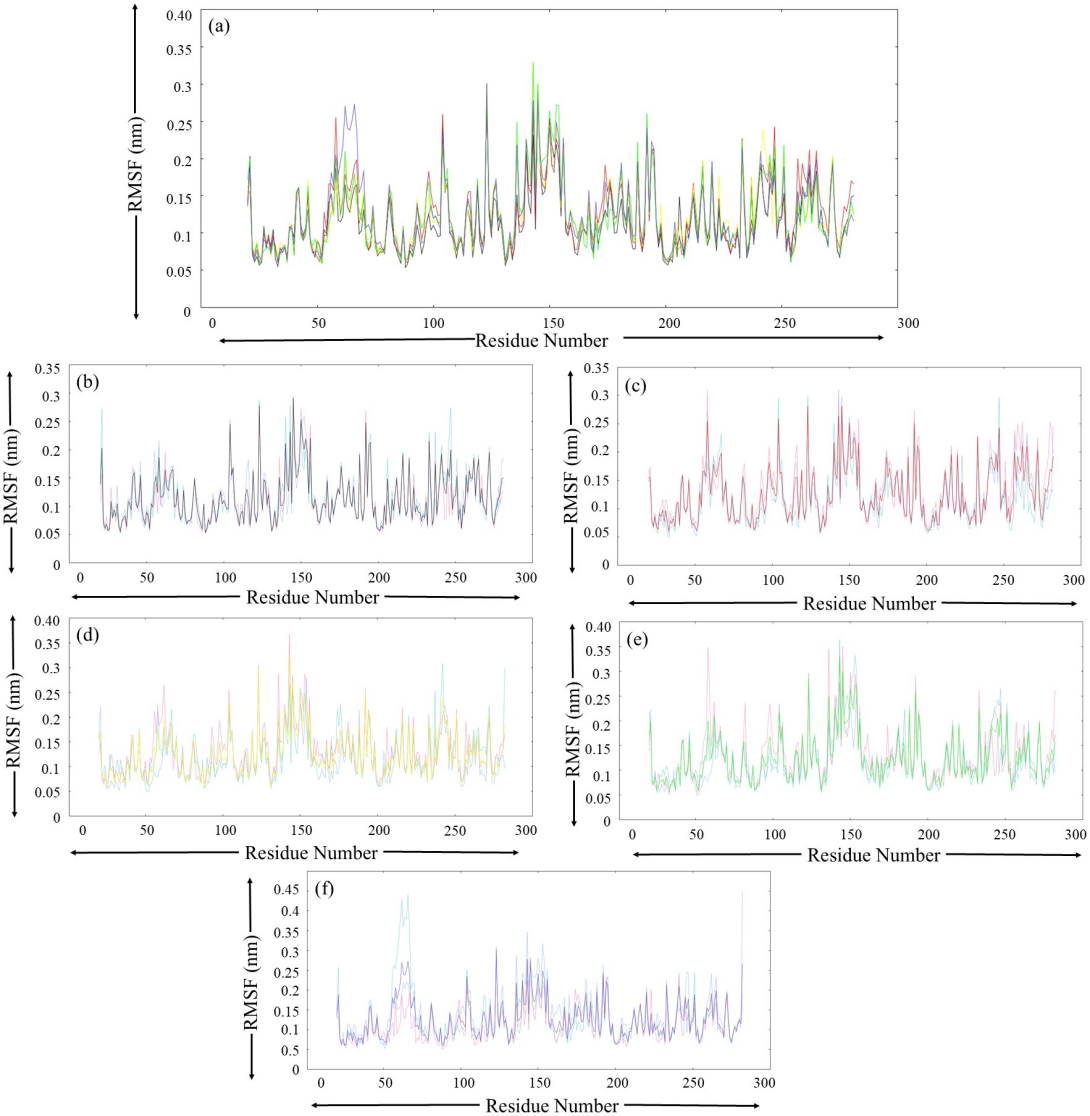
RMSF analysis (a) average RMSF plot for each protein system obtained from three MD simulation runs, colour-coded as black, red, yellow, green, and blue for the following protein system respectively (b) Free-MurI, (c) MurI-Z1542321346, (d) MurI-Z2356864560, (e) MurI- Z2228760853, and (f) MurI- Z3214698999. The RMSF trend for run-1, run-2, and run-3 are represented with turquoise, light blue, and pink colours, respectively.

Next, an RMSF analysis was performed to assess the per-residue fluctuations of MurI and its ligand-bound complexes. The RMSF plot (Fig 5) displayed significant consistency in terms of patterns of fluctuations put forth by each MurI system during the MD simulations performed in triplicates. For native MurI protein, the highest peaks for RMSF were displayed by residues spanning between Lys140-Cys155, and this region was mainly composed of loops. The protein segments consisting of Asn38-Asn46 and Asp54-Ile68 residues also displayed notable fluctuations, which could be attributed to the presence of the loop structures. Still, the RMSF value for these regions remained below 0.23 nm. Apart from these, noteworthy fluctuations were recorded for the residues present in the regions consisting of Trp190-Glu195 and Arg233-Asp250 residues with RMSF values not higher than 0.25 nm. These findings were consistent for all three MD runs with minor variations. The average RMSF values for native MurI obtained from MD triplicates were recorded as 0.116 nm, 0.116 nm, and 0.113 nm for run-1, run-2, and run-3, respectively (Table 6). After visual interpretation of graphs obtained from RMSF analysis for ligand-bound MurI complexes, it was evident that the per residue fluctuations were slightly escalated after ligand binding events. The protein regions consisting of Val114-Asn127, Thr135-Ile157, Leu180-Glu195, and Ala230-Asp250 displayed high fluctuations for all the protein-ligand complexes, and these results were consistent through all three MD runs. The protein segment spanning Thr135-Cys155 residues came out to be highly dynamic since fluctuations were considerably higher for all the MurI systems.

#### 3.5.3. Radius of gyration

**Fig 6:**
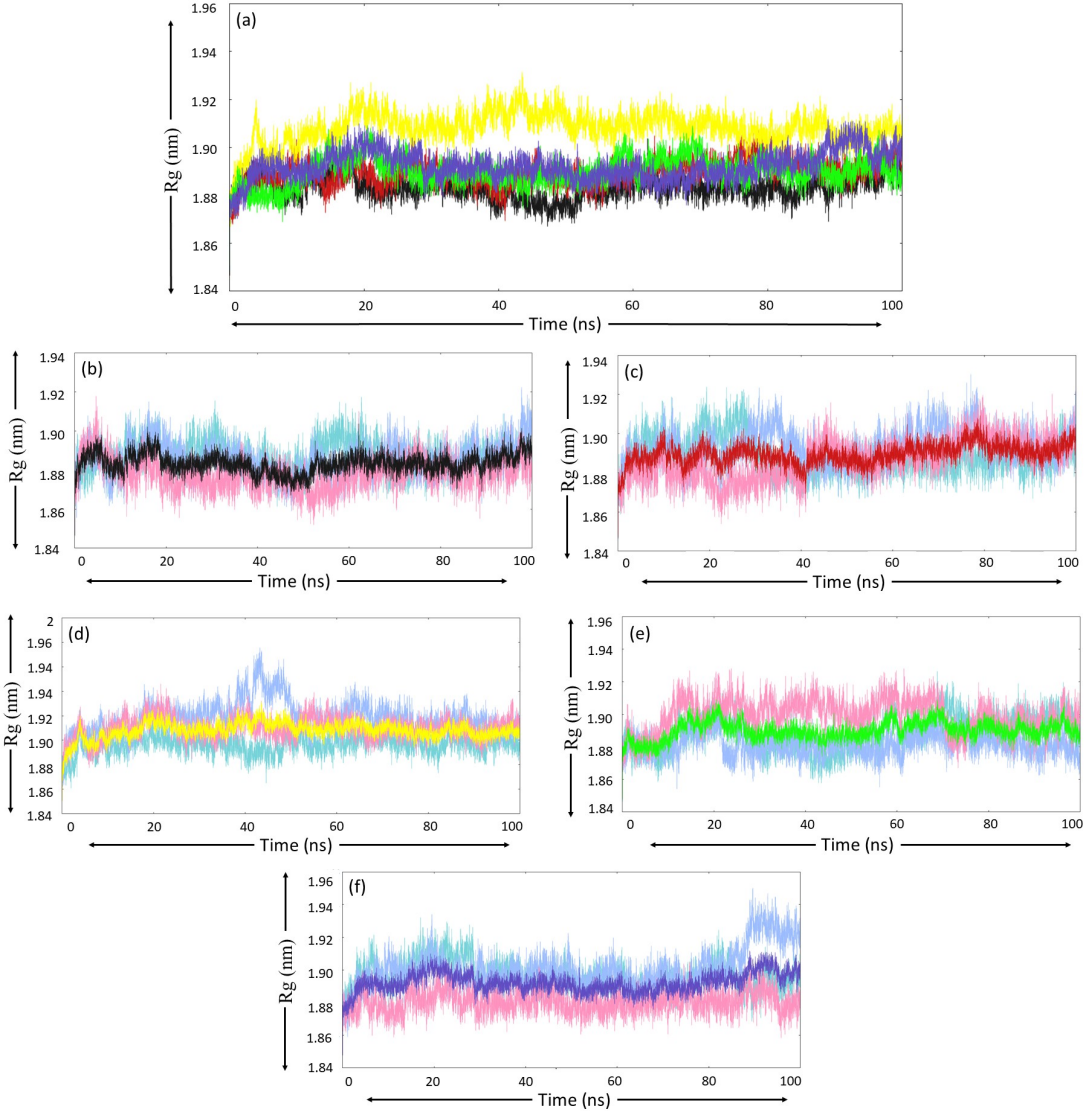
Rg analysis (a) average Rg plot for each protein system obtained from three MD simulation runs, colour-coded as black, red, yellow, green, and blue for the following protein system respectively (b) Free-MurI, (c) MurI-Z1542321346, (d) MurI-Z2356864560, (e) MurI- Z2228760853, and (f) MurI- Z3214698999. The Rg trend for run-1, run-2, and run-3 are represented with turquoise, light blue, and pink colours, respectively.

The Rg plot for native MurI (Fig 6), after visual interpretation, elucidated that trajectories appeared to converge at 70 ns; after that, no to little undulations were observed, and these observations were similar for all MD runs. The value of Rg remained well below 1.90 nm throughout the simulations, and average Rg values for MD triplicates were 1.89 nm, 1.89 nm, and 1.88 nm, further bolstering the consistency of MD simulations. The MurI-Z1542321346 complex, in comparison to native MurI showed more stable trajectories for Rg; the convergence was attained close to 40 ns in run-1 and run-2, whereas the trajectory for run-3, after experiencing slight undulations, finally converged at 80 ns. The average Rg values for this complex were quite similar to the native protein, indicating that the binding of this ligand did not significantly affect the compactness of the protein. For the MurI-Z2356864560 complex, the graph attained convergence after 68 ns in all the runs. The trajectory for run-1 and run-3 exhibited similar patterns throughout the simulation period; however, the graph for run-1 displayed lower Rg values compared to other runs. The average Rg values calculated for each run (Table 6) were also closely similar but higher compared to the native protein and MurI-Z1542321346 complex, suggesting that the binding of Z2356864560 to MurI slightly decreased the protein compactness. The Rg graph for the MurI-Z2228760853 complex displayed an upward trend till 24 ns, hitting a maximum. Beyond this point, the Rg value did not increase, but minor perturbations were observed until 80 ns. After this point, the graph attained convergence and maintained this stable state till the end of the simulation. The MurI-Z3214698999 complex displayed the most stable trajectories among all protein systems. After achieving stability at around 30 ns the graph followed a straight path with minor undulations till 85 ns, after which significant perturbations were seen. The average Rg values of MurI and its complexes are provided in Table 6.

#### 3.5.4. Solvent accessible surface area

**Fig 7:**
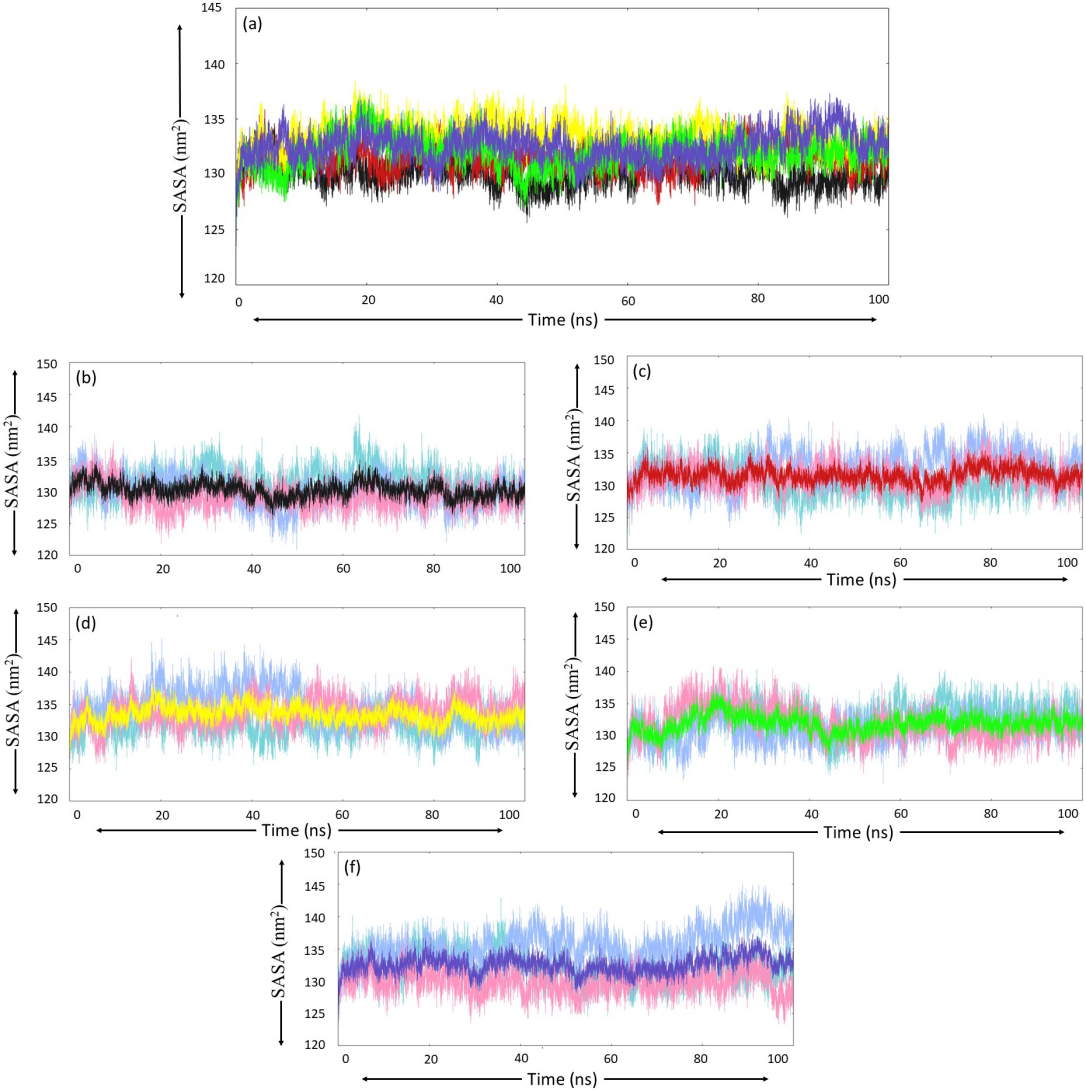
SASA analysis (a) average SASA plot for each protein system obtained from three MD simulation runs, colour-coded as black, red, yellow, green, and blue for the following protein system respectively (b) Free-MurI, (c) MurI-Z1542321346, (d) MurI-Z2356864560, (e) MurI- Z2228760853, and (f) MurI- Z3214698999. The SASA trend for run-1, run-2, and run-3 are represented with turquoise, light blue, and pink colours, respectively.

Next, SASA analysis was performed, which determined the surface area of protein available for interaction with the solvent molecules. The visual interpretation of the SASA plot for free MurI protein revealed that except for run-3, where the plot appeared linear after 25 ns, the other two MD runs displayed significant fluctuations in the graph, indicating the dynamic nature of protein structure. However, The SASA value didn’t go beyond 140 nm^2^, and the calculated average values were also in the range of 129 nm^2^ to 131.5 nm^2^ (Table 6). After the ligand binding events, an increase in the SASA values was observed for all the protein-ligand complexes. The MurI-Z2356864560 and MurI-Z3214698999 complexes displayed the highest SASA values. The other two ligands exhibited closely similar values for SASA, which can be seen in Table 6, and the SASA plot is shown in Fig 7.

**Fig 8:**
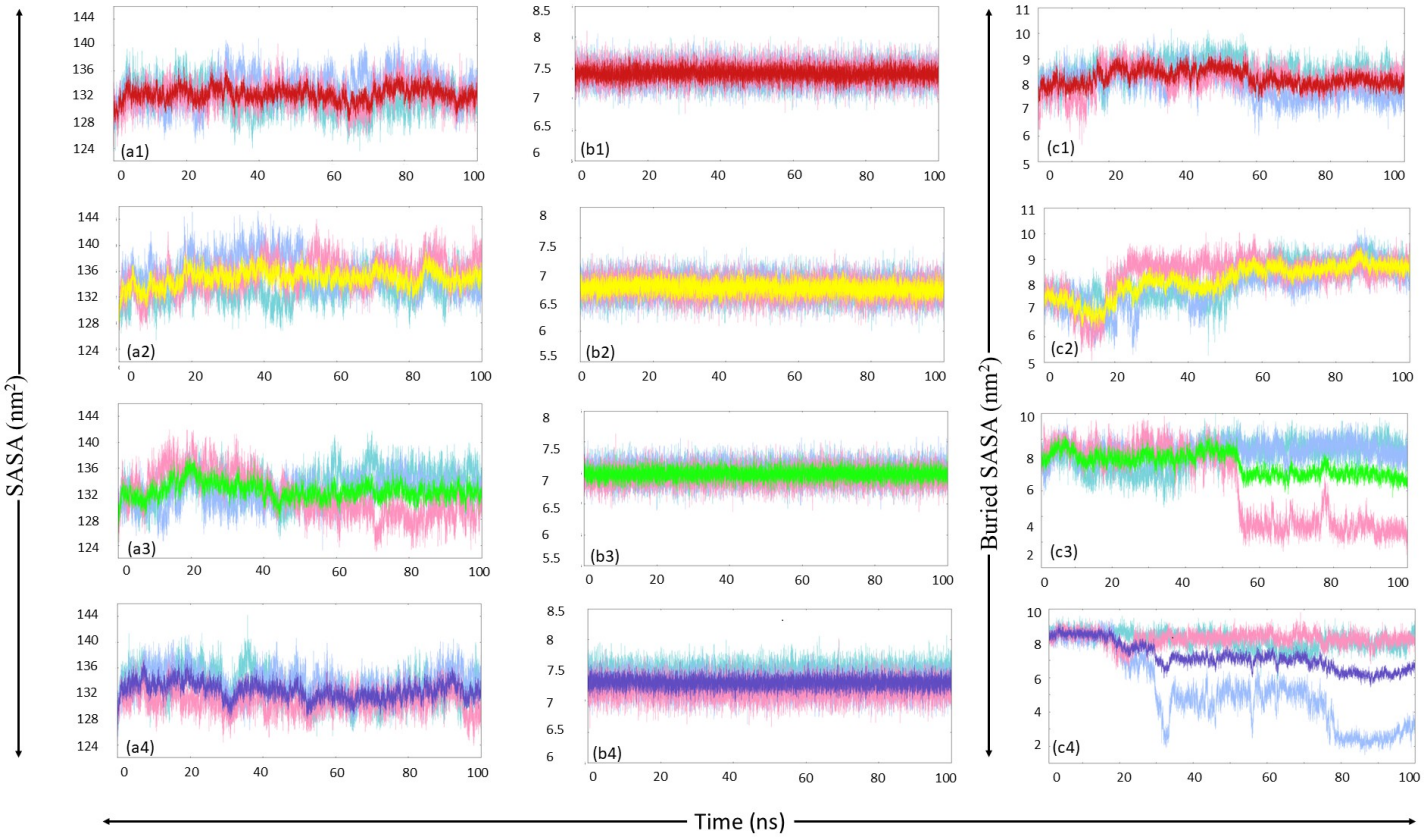
Buried SASA analysis (1) MurI-Z1542321346, (2) MurI-Z2356864560, (3) MurI-Z2228760853, and (4) MurI-Z3214698999; column ‘a’ represents protein SASA, column ‘b’ represents ligand SASA, and column ‘c’ represents buried SASA. The averaged buried SASA plots from three MD runs are coloured red, yellow, green, and blue for Z1542321346, Z2356864560, Z2228760853, and Z3214698999 ligands respectively. The run-1, run-2, and run-3 for each complex are represented with turquoise, light blue, and pink, respectively.

Buried-SASA was also calculated to assess the extent of the binding of ligands to the protein molecule. The buried SASA plot with ligand SASA and protein SASA for each complex is provided in Fig 8. MurI-Z1542321346 complex displayed consistently good values for buried SASA in all three MD runs; the average value from three runs was calculated to be 8.03 nm^2^. The plot for buried SASA for this complex appeared stable without major fluctuations, indicating that the ligand maintained good interaction with the protein throughout the simulation period across all three runs. MurI-Z2356864560 complex displayed the best buried SASA score among four ligands; the average value for three runs was 8.15 nm^2^. The graph for the analysis of this complex exhibited initial fluctuations. Still, it stabilized after 35 ns and maintained stability for the rest of the simulation. These observations were consistent for MD triplicates, suggesting that the interaction of this ligand was stable and robust with the protein.

The average value of buried SASA for the MurI-Z2228760853 complex was calculated as 8.04 nm^2^, similar to MurI-Z1542321346, but the buried SASA plot displayed slight inconsistency during the three runs. Despite showing significant stability in graphs for run-1 and run-2, the graph for run-3 appeared quite unstable. The graph experienced a sudden dip after 50 ns and reached a value of 4 nm^2^, and the value remained the same for the rest of the simulation period. It could be deduced from these observations that the ligand displayed good interaction with the protein, but the interaction was not consistently strong; possibly, the ligand was moving out of the binding pocket. Similar results were seen for the MurI-Z3214698999 complex, which also exhibited an anomalous graph for run 3. Just like the last discussed complex, a sudden dip after 32 ns was observed, and after this point, the graph displayed exceptionally high fluctuations and low buried SASA values. The values of buried SASA, protein SASA, and ligand SASA are provided in Table 7 for all protein-ligand complexes.

**Table 7:** Buried SASA values of protein-ligand complexes for MurI protein.

System	SASA_protein_ (nm^2^)	SASA_ligand_ (nm^2^)	SASA_complex_ (nm^2^)	SASA_buried_ (nm^2^)
MurI-Z1542321346				
run-1	131.47	7.41	130.33	8.08
run-2	133.41	7.40	132.82	7.99
run-3	132.07	7.43	131.28	8.02
average	132.32	7.41	131.48	8.03
MurI- Z2356864560				
run-1	133.39	6.70	131.92	8.17
run-2	135.80	6.70	134.63	7.87
run-3	135.50	6.69	133.78	8.41
average	134.92	6.69	133.44	8.15
MurI-Z2228760853				
run-1	133.63	7.50	132.78	8.35
run-2	132.04	7.51	131.01	8.54
run-3	131.71	7.46	131.92	7.25
average	132.46	7.48	131.90	8.04
MurI-Z3214698999				
run-1	132.97	7.46	132.24	8.19
run-2	133.51	7.26	135.58	5.19
run-3	130.89	7.20	129.81	8.27
average	132.46	7.30	132.54	7.22

#### 3.5.5. Principal component analysis

**Fig 9:**
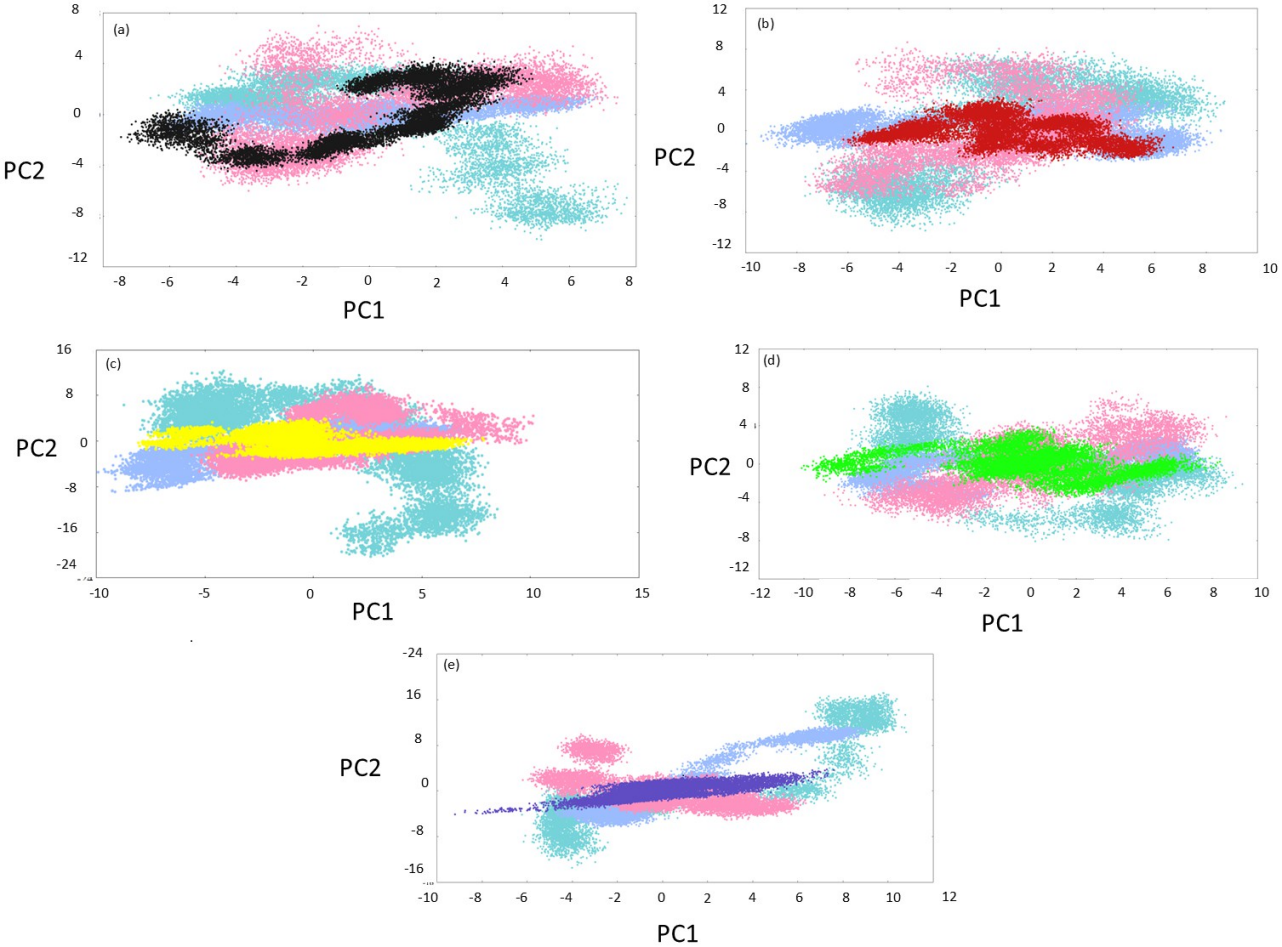
PCA analysis for (a) Free-MurI, (b) MurI-Z1542321346, (c) MurI-Z2356864560, (d) MurI-Z2228760853, and (e) MurI-Z3214698999. The averaged PCA plots from three MD runs are coloured black (free protein), red (MurI-Z1542321346 complex), yellow (MurI-Z2356864560 complex), green (MurI-Z2228760853), and blue (MurI-Z3214698999). The run-1, run-2, and run-3 for each protein system are represented with turquoise, light blue, and pink colours, respectively.

PCA is a dimensionality reduction technique that linearly transforms a trajectory’s feature vectors, producing eigenvectors representing the variance in the data as the motion (or coordinates) of the Cα atoms in the protein molecule. The principal components (PCs) derived from PCA are plotted onto a conformational subspace that illustrates the variances in atomic positions, representing the protein conformation. This is referred to as the essential subspace. The PCA plot (PC1 vs PC2) (Fig 9) for native MurI appeared substantially distributed through the essential subspace. For run-1 and run-3, despite the dispersed appearance of the PCA graphs, the maximum number of conformations was confined to smaller regions where dense clusters could be seen. In run-3, dense, closely spaced clusters were not observed, but the graph was less dispersed. Visual interpretation of the PCA plot suggested that the space occupied by protein conformations in essential subspace for all three MD runs was closely similar. These observations indicated that the free protein successfully achieved low energy states, and maximum conformations attained by the protein resembled those stable states, only indicating good stability and low structural variance. The PCA plot for the MurI-Z1542321346 complex revealed that despite the variability in the acquired subspace for the graphs from MD triplicates, there were common overlapping regions in the essential subspace for the three MD runs. These regions are distinctly conspicuous on the PCA plot of this complex; again, dense clusters of conformations were observed in the plot, few of which were common in all three runs. These overlapping regions could represent relevant conformations attained by the protein-ligand complex.

The MurI-Z2356864560 complex produced interesting results on the PCA plot, and both run-1 and run-2 exhibited exceptional similarity in the graph pattern. The graph for both runs shared larger overlapping regions in the essential subspace, indicating low conformational variance. The PCA graph for the third run was confined in terms of the area occupied in the essential subspace, and some overlapping regions were shared with other runs.

MurI-Z2228760853 also displayed similar results as the previous complex, where run-1 and run-3 exhibited significant similarity in the PCA graph while run-2 showed slight anomalous behaviour. Also, the PCA plot appeared visually more scattered for all MD runs, indicating relatively high conformational variability. MurI-Z3214698999 complex also displayed similar patterns for two runs (run-1 and run-3), while run-2 displayed a more dispersed graph for PCA and three distinct dense clusters were seen for this run.

### 3.6. MM/PBSA-based binding free energy calculations

**Fig 10:**
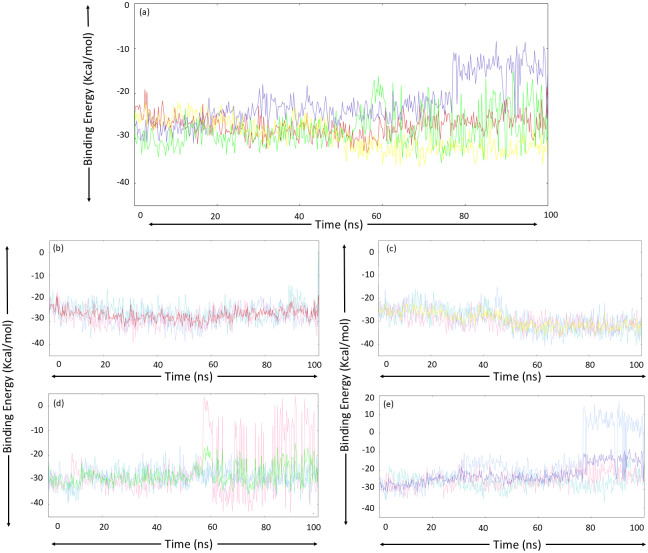
Binding free energy analysis (a) average Binding free energy plot for each protein system obtained from three MD simulation runs, colour-coded as red, yellow, green, and blue for the following protein system respectively (b) MurI-Z1542321346, (c) MurI-Z2356864560, (d) MurI- Z2228760853, and (e) MurI- Z3214698999. The Binding free energy trends for run-1, run-2, and run-3 for each complex are represented with turquoise, light blue, and pink colours, respectively.

Next, binding free energy was calculated using the MM/PBSA-based approach and utilizing the *g_mmpbsa* module. The contributing energies taken into consideration for the calculation of binding free energy are Van der Waals energy, electrostatic energy, polar solvation energy, and SASA energy, and the calculated values of these energies, along with binding free energy, are provided in Table 8. The entire 0–100 ns trajectories were analyzed using MM/PBSA, and the graphs of binding free energy obtained through this analysis are presented in Fig 10. The MM/PBSA analysis was conducted three times for each complex to increase the reliability and robustness. All the complexes showed satisfactory binding free energy values across all three MD runs. The MurI-Z2356864560 complex exhibited the highest binding free energy values, which were also more consistent across the triplicates. However, the MurI-Z1542321346 and MurI-Z2228760853 complexes demonstrated the highest stability and the lowest standard deviation in terms of binding free energy. The MurI-Z3214698999 complex offered the lowest values for this analysis, and the variability in the results was relatively higher for this complex. The average binding free energy values calculated by taking mean of energies obtained from each run were as follows: -27.26±3.06 kcal/mol, -29.53±4.29 kcal/mol, -28.20±5.9 kcal/mol, and -22.26±6.89 kcal/mol for MurI-Z1542321346, MurI-Z2356864560, MurI-Z2228760853, MurI-Z3214698999 complexes respectively. These values suggested that the ligand molecules had considerably strong binding with the targeted site of the MurI protein.

**Table 8:** The binding free energy values and other contributing energy components responsible for complex formation between protein and selected ligands.

System	Van der Waals energy (kcal/mol)	Electrostatic energy (kcal/mol)	Polar solvation energy (kcal/mol)	SASA energy (kcal/mol)	Binding energy (kcal/mol)	Average Binding energy for three runs (kcal/mol)
MurI-Z1542321346						
run-1	-39.75±3.68	-5.51±1.95	23.78±4.81	-4.58±0.30	-26.07±3.91	-27.26±3.06
run-2	-35.47±3.20	-2.63±2.82	15.00±5.18	-4.27±0.32	-27.28±4.10	
run-3	-38.30±3.78	-4.30±3.16	18.56±6.14	-4.40±0.31	-28.44±1.18	
MurI-Z2356864560						
run-1	-39.684±6.41	-1.14±1.55	15.54±4.95	-4.42±0.48	-29.71±4.88	-29.53±4.29
run-2	-39.27±6.27	-1.97±1.41	16.76±4.79	-4.30±0.49	-28.78±4.54	
run-3	-41.63±4.58	-1.80±1.27	17.93±4.14	-4.54±0.46	-30.09±3.46	
MurI-Z2228760853						
run-1	-37.44±3.00	-2.15±1.38	15.25±3.64	-4.40±0.30	-28.74±3.64	-28.20±5.9
run-2	-39.87±2.81	-3.19±1.43	18.59±3.40	-4.55±0.22	-29.02±3.40	
run-3	-34.24±4.80	-2.11±1.22	13.34±10.22	-3.85±0.77	-26.86±10.66	
MurI-Z3214698999						
run-1	-34.69±4.41	-3.38±2.03	16.39±6.17	-4.36±0.30	-26.05±3.67	-22.26±6.89
run-2	-20.99±11.42	-3.98±1.84	11.49±8.93	-2.68±1.13	-16.17±12.88	
run-3	-39.36±3.62	-2.89±2.50	22.10±4.06	-4.43±0.25	-24.57±4.13	

## 4. Discussion

In the current study, the MurI (glutamate racemase) protein from *Klebsiella pneumoniae* was put under scrutiny, and the primary focus was to search for ligands which could act as candidate inhibitors against this protein. First, the three-dimensional structure of MurI was modelled using SWISS-MODEL, for which MurI from *E*. *coli* was taken as a template. The modelled structure was then subjected to virtual screening using the TACC server against the Enamine AC library. TACC server brought forth 1000 molecules with the highest estimated binding affinities towards the targeted site of the protein. Out of 1000, four molecules were selected for further studies after getting screened through Lipinski’s Rule of Five and ADME parameters. The protein-ligand complexes of these four ligands were then subjected to MD simulations for a period of 100 ns, and the simulations were performed in triplicates. The MD trajectories were then used to perform various analyses such as RMSD, RMSF, Rg, SASA, buried-SASA, and PCA.

The RMSD plot for native protein displayed stable trajectories throughout the simulation period across all three MD runs; small perturbations were seen before 50 ns, but RMSD values were quite stable afterwards. Similarly, SASA values also stabilized after 50 ns, and very low changes were observed after this point in time. These findings were further supported by PCA, where despite dispersed plots, dense and distinct conformational clusters were observed, indicating that maximum conformations attained by protein had low variance. These observations clearly indicated that the protein molecule displayed good stability and low conformational variability during the MD simulations. RMSF analysis revealed that the regions corresponding to Ala122-Leu124, Lys140-Cys155, Trp190-Glu195, and Arg233-Phe255 residues displayed high magnitude fluctuations, which could be attributed to the presence of loop structures.

The MurI-Z1542321346 complex, like native protein, displayed stable RMSD values. The graph for RMSD for the complex showed convergence after 30 ns, and very low fluctuations were observed after this point. The RMSD values were a little higher than native protein, indicating that the binding of Z1542321346 to protein increased the dynamic behaviour of the protein molecule. The Rg graphs appeared perturbed for the initial 45 ns and maintained a stable path for the rest of the simulation period; however, some perturbations were seen at 80 ns in all three runs. This complex displayed slightly higher Rg values in comparison to the protein supporting the RMSD results. The SASA analysis also exhibited higher values for this complex in relation to native protein, offering a stable graph with minor perturbations. The findings of SASA and Rg analyses suggested that the compactness of the protein decreased after binding to the Z1542321346 molecule. The complex offered higher RMSF values compared to native protein, indicating increased motion in the protein structure after ligand binding. This molecule exhibited strong binding affinity and high binding free energy values from Autodock Vina-based docking and MM/PBSA-based binding free energy analysis, values for which are -8.5 kcal/mol and -27.26±3.06 kcal/mol, respectively. Buried-SASA analysis results also supported the notion of strong binding of Z1542321346 to the protein.

The RMSD graph of the MurI-Z2356864560 complex for run-2 was highly perturbed and acquired exceptionally high values throughout the simulation. The other two runs also exhibited high RMSD values in relation to free protein, but less perturbations were there. The Rg and SASA analysis displayed coherence with the RMSD results, as both of these analyses suggested that the protein compactness decreased even more upon binding of Z2356864560. The complex exhibited higher values of SASA and Rg among the three protein systems discussed so far. The PCA analysis suggested that the conformational variability was low for this complex. The dense clusters on the essential subspace and overlapping regions among plots for different runs suggested that the protein system did acquire stable states, and the majority of conformations existed in those states, only supporting the stability of the MurI-Z2356864560 complex. The binding affinity value calculated using Autodock Vina for this molecule was -8.5 kcal/mol. Impressive binding free energy value (-27.26±4.29 kcal/mol) with low standard deviation also indicated strong binding of the ligand to the protein molecule, which was congruent with the findings of the buried-SASA analysis. After displaying fluctuations, the buried SASA plot for the protein system initially acquired stability around 60 ns and maintained a decent buried SASA value for the rest of the simulation period. The complex displayed lower average RMSF values than the MurI-Z1542321346 complex, but the values were higher in comparison to those of the native protein. The regions close to the hinge region displayed the highest peaks in the RMSF plot, suggesting increased motion of the two domains of the protein.

The MurI-Z2228760853 complex also exhibited similar values of RMSD when compared to native protein; the fluctuations in the RMSD plot were not very high, and the trajectories seemed to converge after 75 ns for all three runs. The average RMSD from three runs was similar to native protein. The Rg values of this complex were lower than the two complexes discussed so far; also, the Rg plot resembled to that of free protein, suggesting that the compactness of the protein was majorly unaffected by the binding of this molecule. These observations corroborate the findings of SASA analysis, which also indicated that the protein remained compact and was dynamically less active. The PCA plot was also relatively more compact than other protein systems, supporting the high conformational stability of the complex. The complex assumed a high buried SASA value for two md runs (run-1 and run-2). However, the third run proved to be anomalous to this trend, displaying sudden dip in the buried SASA after 55 ns and the value remained low for the rest of the simulation period. These observations were in congruence with the results of MM/PBSA-based binding free energy. Despite having exceptionally high binding free energy values for two runs, the plot for the third run appeared surprisingly perturbed after 55 ns, lowering the average binding free energy value for this complex. The average RMSF value for this complex from three MD runs was similar to that of MurI-Z2356864560, and there was also a significant similarity in the pattern of fluctuations in the RMSF plot. Run-3 displayed the highest fluctuations among triplicates for this complex, corroborating the buried SASA findings and binding free energy analysis.

The MurI-Z3214698999 complex, despite early convergence around 25 ns, displayed slightly high RMSD values throughout the simulation. The RMSD values for run-3 further heightened after 85 ns and continued to do so for the remaining simulation period. These observations were in sync with the findings of Rg and SASA analyses; despite maintaining the low values in two MD runs, the graphs for the second run displayed high values with significant fluctuations towards the end for both analyses. The buried SASA plot was quite stable and had high buried SASA values for run-1 and run-3, but the graph for run-2 appeared exceptionally perturbed and exhibited surprisingly low values. These observations did not support this complex’s stable behaviour, especially in run-2. The binding free energy graph was also in congruence with other analyses. High fluctuations were seen for run-2 for the last 20 ns of the simulation period, but the complex displayed decent binding free energy values in the other two runs. The RMSF analysis also offered similar findings where run-2 exhibited exceptionally high RMSF peaks.

The RMSD plot offered the lowest values for native protein among all protein systems, followed by MurI-Z2228760853 and MurI-Z1542321346 complexes. The MurI-Z2356864560 complex has the highest RMSD values to offer among all complexes. Similar trends were observed for Rg and SASA plots also, where MurI-Z2228760853 and MurI-Z1542321346 complexes had lower values after native protein, and the MurI-Z2356864560 had the highest values. Buried SASA values were considerably high for MurI-Z1542321346 and MurI-Z2356864560 complexes in comparison to the other two complexes, indicating good interaction with the protein molecule. The Binding free energy values, particularly for these two complexes, were exceptionally good with low standard deviation; the values were recorded as -27.26±3.06 kcal/mol and -29.53±4.29 kcal/mol for MurI-Z1542321346 and MurI-Z2356864560 complexes respectively. The ligands Z1542321346 and Z2356864560 were seen to be interacting with the similar residues of the cryptic site of the MurI protein as described by Chheda et al. in their study focused on MurI from *H*. *pylori*. They discussed the interaction pattern of the NP-020560 molecule along with binding affinity values and proposed it as a good candidate for allosteric inhibition of glutamate racemase [23]. The molecules proposed in our study showed a similar interaction pattern to this ligand, and the interaction shown by these molecules with the targeted site of the protein is also strong.

## 5. Conclusion

The current study focuses on targeting the MurI (glutamate racemase) protein of *Klebsiella pneumoniae* to identify potent inhibitors that could mitigate the pathogen’s virulence. Utilizing homology modelling via SWISS-MODEL and using *E*. *coli* MurI as a template, we successfully modelled the 3D structure of *Klebsiella pneumoniae* MurI. This model was subjected to virtual screening against the Enamine AC library using the TACC server, which identified 1000 potential molecules with high binding affinities. Further screening based on Lipinski’s Rule of Five and ADME parameters narrowed the candidates down to four promising ligands.

Molecular dynamics (MD) simulations conducted over 100 ns for the four protein-ligand complexes revealed insights into their stability and the binding efficiency of the ligands to the protein molecule. The analyses such as RMSD, Rg, SASA, buried-SASA, and PCA indicated varying degrees of stability and compactness upon binding of the ligands to the protein. The native protein exhibited stable RMSD, Rg, and SASA values, signifying a stable structure throughout the three MD simulation runs. The MurI-Z1542321346 complex demonstrated stable RMSD values post 30 ns, though it slightly increased the protein’s dynamic behaviour. The Rg and SASA analyses showed that protein compactness was slightly lower for this complex when compared to native protein, but the plots for these analyses were quite stable, and these findings were supported by strong binding affinity and high binding free energy values. The MurI-Z2356864560 complex showed high RMSD values, with some perturbations, but maintained stable states as indicated by PCA. This complex also had the highest Rg and SASA values, suggesting a significant decrease in compactness upon ligand binding. Despite fluctuations, its binding free energy remained impressively high, indicating strong protein-ligand interaction.

The MurI-Z2228760853 complex exhibited RMSD and Rg values comparable to the native protein, indicating minimal impact on compactness and dynamic behaviour. The binding free energy analysis and SASA results supported these findings, though one MD run showed anomalous behaviour. The MurI-Z3214698999 complex displayed early RMSD convergence but had slightly higher RMSD values and significant fluctuations towards the end of one MD run, correlating with its Rg and SASA analysis results. This complex’s binding free energy values varied greatly across MD triplicates, which was a consequence of high fluctuations in one MD run.

Overall, Z1542321346 and Z2356864560 emerged as the most promising lead candidates, exhibiting strong binding affinities, good stability, and favourable binding free energy values. These findings suggest that targeting MurI with these molecules could be a viable strategy to reduce *Klebsiella pneumoniae’s* virulence and resistance, providing a basis for future drug development efforts.

## Supporting information

S1 Table The calculated allergenicity score for the molecules proposed as candidate drug molecules. (DOCX)

## Abbreviations

ADME: Absorption Distribution Metabolism Excretion
MD: Molecular Dynamics
MDR: Multi-drug resistant
MM/PBSA: Molecular mechanics/Poisson–Boltzmann surface area
PCA: Principal Component Analysis
Rg: Radius of Gyration
RMSD: Root Mean Square Deviation
RMSF: Root Mean Square Fluctuations
SASA: Solvent Accessible Surface Area
UMAG: UDP-N-acetylmuramoyl-L-alanine-D-glutamate
