# Supplementary material for: Identification and prioritization of novel therapeutic candidates against glutamate racemase from Klebsiella pneumoniae
Source: PLoS One. 2025 Feb 6;20(2):e0317622. doi: 10.1371/journal.pone.0317622 (PMC11801594; doi:10.1371/journal.pone.0317622)
Supplement: S1 Table — (DOCX) [file pone.0317622.s001.docx]

**Supporting Information**

**Identification and prioritization of novel therapeutic candidates against Glutamate racemase from *Klebsiella pneumoniae***

Ankit Kumar^1^, Farah Anjum^2^, Md Imtaiyaz Hassan^3^, Anas Shamsi^4,^*

Rashmi Prabha Singh^5,^*

^1^Department of Biotechnology, Sharda School of Engineering and Technology, Sharda University, P.C. 201310, Greater Noida, U.P., India

^2^Department of Clinical Laboratory Sciences, College of Applied Medical Sciences, Taif University, PO Box 11099, 21944, Taif, Saudi Arabia.

^3^Centre for Interdisciplinary Research in Basic Sciences, Jamia Millia Islamia, Jamia Nagar, New Delhi 110025, India

^4^Centre of Medical and Bio-Allied Health Sciences Research, Ajman University, Ajman 364, United Arab Emirates.

^5^ Department of Life Science, Sharda School of Basic Sciences and Research, Sharda University, P.C. 201310, Greater Noida, U.P., India

***Corresponding Author:** Dr. Rashmi Prabha Singh, E-mail: [rashmi.singh@sharda.ac.in](mailto:rashmi.singh@sharda.ac.in)

Co-corresponding Author: Dr. Anas Shamsi, Email: anas.shamsi18@gmail.com

**Manuscript ID:** PONE-D-24-36098

**S1 Table:** The calculated allergenicity score for the molecules proposed as candidate drug molecules.

| **The allergenicity score for the selected Ligands** | | | |
| --- | --- | --- | --- |
| **S.No.** | **Molecule** | **Score** | **Prediction** |
| 1. | Z1542321346 | 0.25 | Non-allergen |
| 2. | Z2356864560 | 0.15 | Non-allergen |
| 3. | Z2228760853 | 0.16 | Non-allergen |
| 4. | Z3214698999 | 0.15 | Non-allergen |

***Score Under 0.35 indicates unlikely sensitization to the allergen**

**Reference:**

Sharma N, Patiyal S, Dhall A, Devi NL, Raghava GP. ChAlPred: a web server for prediction of allergenicity of chemical compounds. Computers in Biology and Medicine. 2021 Sep 1;136:104746.
